# A rare and severe complication in a patient who underwent percutaneous endoscopic lumbar discectomy: A case report

**DOI:** 10.1097/MD.0000000000048292

**Published:** 2026-04-17

**Authors:** Yuxiang Wang, Yanyu Qi, Rong Huang, Xiangjing Kong, Shoushi Wang

**Affiliations:** a Department of Anaesthesia and Perioperative Medicine, Qingdao Central Hospital, University of Health and Rehabilitation Sciences (Qingdao Central Medical Group), Qingdao, Shandong Province, China; b Qingdao Medical College, Qingdao University, Qingdao, Shandong Province, China.

**Keywords:** dural tear, intracranial hypertension, irrigation pressure, lumbar discectomy

## Abstract

**Rationale:**

Percutaneous endoscopic lumbar discectomy is widely performed under general anesthesia. Acute intracranial hypertension caused by massive cerebrospinal fluid loading via an unrecognized dural tear is an under-reported, potentially fatal complication that may be masked by anesthesia.

**Patient concerns:**

A 64-year-old woman underwent elective L3–L4 endoscopic discectomy. Intraoperatively, she developed refractory hypertension, followed by generalized tonic-clonic seizures and altered consciousness shortly after extubation.

**Diagnoses:**

Intraoperative dural tear with retrograde irrigation-fluid influx causing acute intracranial hypertension.

**Interventions:**

Immediate cessation of irrigation, 20° head-up positioning, mannitol 20% 200 mL intravenous (IV), dexmedetomidine 0.7 µg kg^−1^ h^−1^, propofol 100 mg IV, and methylprednisolone 80 mg IV.

**Outcomes:**

Irrigation-related intracranial hypertension was promptly managed; seizures stopped within 45 minutes, and full consciousness (Glasgow Coma Scale 15) was restored by 4 hours. Hemodynamics and arterial blood gas normalized without extra antihypertensives. No further sedation or antiepileptics were needed. At 24 hours, the National Institutes of Health Stroke Scale was 0, the Mini-Mental State Examination score was 29/30, and no meningeal signs. Assisted mobilization on postoperative day (POD) 2 and independent ambulation on POD 5. Discharged on POD 7 with modified Rankin Scale 0 and no symptoms; 30-day follow-up confirmed modified Rankin Scale 0 and no neurological sequelae.

**Lessons:**

Refractory hypertension during percutaneous endoscopic lumbar discectomy should prompt immediate consideration of cerebrospinal fluid hypertension due to dural breach. Early recognition and prompt intervention are critical to prevent permanent neurological damage.

## 1. Introduction

Percutaneous endoscopic lumbar discectomy (PELD) has gained widespread acceptance for the treatment of lumbar disc herniation owing to its minimal tissue trauma, reduced postoperative pain, and accelerated recovery.^[1]^ Continuous pressurized irrigation is indispensable for maintaining a clear operative field and achieving hemostasis; however, it introduces a unique spectrum of complications, including nerve root injury, residual disc fragments, hematoma, and dural tears.^[2]^

Among these, acute intracranial hypertension secondary to massive cerebrospinal fluid (CSF) volume loading through an iatrogenic dural breach is rarely reported and may be catastrophic.^[3]^ When surgery is performed under general anesthesia, early clinical signs – namely, hypertension, bradycardia, or agitation – are frequently attributed to inadequate anesthesia depth rather than to a surgical complication, thereby delaying diagnosis.

The entity has been variably labeled “myeloid hypertension-like syndrome” in Chinese literature.^[4]^ Yet, this term lacks pathophysiological precision and risks confusion with venous hypertensive myelopathy (VHM), a chronic veno-occlusive condition. We report a case in which rapid recognition of irrigation-related CSF hypertension and targeted intervention prevented permanent neurological injury.

## 2. Case presentation

### 2.1. Clinical history

**Figure 1.**
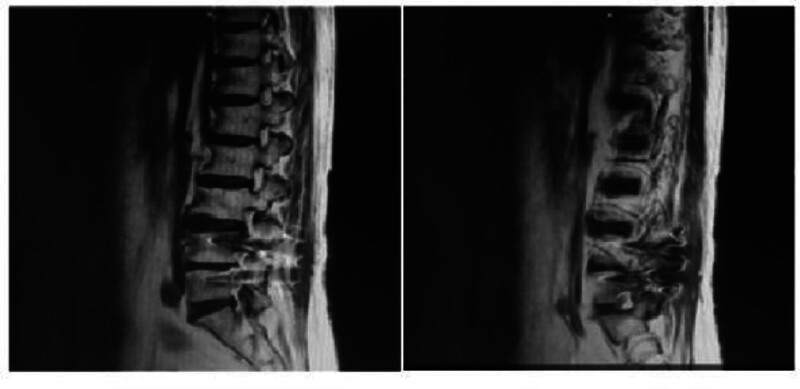
Preoperative magnetic resonance imaging revealed recurrent disc herniation at L3/4 as the primary pathological segment, with circumferential disc bulging noted from L1/2 to L5/S1.

A 64-year-old woman (165 cm, 78 kg) presented with 8-week low-back pain radiating to the left leg. She denied any visual disturbances, headache, or prior neurological deficits. Past medical history included hypertension (valsartan-controlled), paroxysmal atrial fibrillation, and prior posterior lumbar instrumentation (L4–S1) 7 years earlier. No intracranial pathology was documented on prior imaging. Preoperative magnetic resonance imaging revealed current disc herniation at L3/4 and circumferential bulging from L1/2 to L5/S1 (Fig. 1). No intracranial pathology was documented on prior imaging.

Routine laboratory tests, including complete blood count, renal and hepatic panels, coagulation profile, and serum electrolytes, were within normal limits. Preanesthetic airway assessment was unremarkable (Mallampati I, mouth opening 3 fingerbreadths, neck extension >35°).

### 2.2. Perioperative course, surgical events, and postoperative course

Upon arrival in the operating theater, baseline noninvasive blood pressure was 135/75 mm Hg, heart rate 62/min, and SpO_2_ 99%. Following application of standard monitors, a left radial arterial catheter was inserted under local anesthesia for continuous arterial pressure monitoring. Anesthesia was induced with intravenous lidocaine 50 mg, sufentanil 15 µg, propofol 100 mg, etomidate 8 mg, and rocuronium 50 mg. A 7.0-mm cuffed endotracheal tube was placed under video laryngoscopy. Maintenance consisted of sevoflurane 1% to 3% in oxygen/air and remifentanil 0.1 to 0.2 µg kg^−1^ min^−1^. Dexamethasone 5 mg intravenous (IV) and ondansetron 8 mg IV were administered for antiemesis.

**Figure 2.**
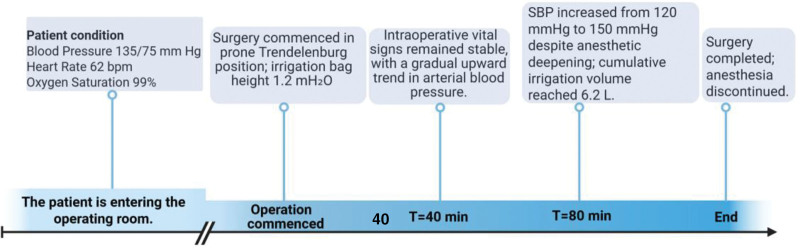
Preoperative and intraoperative timeline. SBP = systolic blood pressure.

At the commencement of surgery, the patient was placed in a prone Trendelenburg position with the irrigation bag set at a height of 1.2 mH_2_O (≈90 mm Hg hydrostatic pressure). Forty minutes later, intraoperative vital signs remained stable, although arterial blood pressure showed a gradual upward trend. At 80 minutes, systolic blood pressure (SBP) acutely rose from 120 to 150 mm Hg despite the administration of 10 µg sufentanil and a concomitant increase in sevoflurane concentration; irrigation continued, bringing the total irrigation volume to 6.2 L. Upon conclusion of the procedure, estimated blood loss was 20 mL, and sevoflurane was discontinued (Fig. 2). Intraoperative blood pressure and heart rate fluctuations with corresponding management measures are presented in Table 1.

**Table 1:** Changes in blood pressure, heart rate, and respiratory rate at 4 time points – entry into the OR, 40 and 80 minutes after incision, and the end of surgery – together with the concomitant adjustments of sevoflurane, remifentanil, and rocuronium, providing a concise overview of intrahemodynamic stability and anesthetic management.

Time point	Blood pressure (mm Hg)	Heart rate (bpm)	Respiratory rate (breaths/min)	Management
Enter the operating room	135/75	62	Within normal limits	Preanesthesia monitoring
40 min after surgery starts	150/75	Within normal limits	Within normal limits	Sevoflurane 1%–3%, remifentanil 0.1–0.2 μg·kg^−1^·min^−1^, rocuronium 10 mg (IV)
80 min after surgery starts	150/75	Within normal limits	Within normal limits	Increased sevoflurane to 2.5%, adjusted remifentanil to 320 μg/h
End of surgery	140/80	90	20	Extubation

IV = intravenous, OR = operating room.

Seven minutes after tracheal extubation, the patient exhibited generalized tonic-clonic seizures, SBP 155 mm Hg, heart rate 120/min, and respiratory rate 30 min^−1^. Arterial blood gas revealed metabolic acidosis (pH 7.21, lactate 10.8 mmol/L, base excess −13.2 mmol/L) without hypoxemia (Table 2). Serial arterial blood gases show rapid correction of severe mixed acidosis (pH 7.21 → 7.53) and lactate (10.8 → 4.4 mmol/L) within 3 hours. Management was stepwise sedation (propofol + dexmedetomidine), dehydration (mannitol/furosemide), steroids, and oxygen; agitation and convulsions resolved while PaCO_2_ fell from 34 to 27 mm Hg. The detailed arterial blood gas findings and corresponding interventions are provided in Table 2.

**Table 2:** The patient’s postoperative blood gas changes and the physicians’ corresponding management measures.

Time point	Arterial blood gas analysis				Clinical manifestation	Management
	PaCO_2_ (mm Hg)	pH	Lac (mmol/L)	BE (B)		
7 min after tracheal extubation	34	7.21	10.8	−13.2	Agitated → comatose, BP 150/75, HR 120, leg twitching, Kussmaul; post-propofol 100 mg still unconscious with impending doom	Sedation with propofol 100 mg, followed by dexmedetomidine 60 μg and propofol 150 mg, methylprednisolone 2 doses of 80 mg, 20% mannitol 150 mL, and furosemide 20 mg with oxygen therapy
37 min after tracheal extubation	31	7.30	9.0	−10.0	The patient became agitated again	Approximately 50 mL of 20% mannitol was infused, and the infusion of propofol and dexmedetomidine was continued
60 min after tracheal extubation	25	7.47	7.2	−4.0	The patient was in a sedated state	The infusion of 20% mannitol was discontinued, while the infusions of propofol and dexmedetomidine were continued
Approximately 3 h after tracheal extubation	27	7.53	4.4	0.9	The patient remained unconscious and agitated, with a decrease in the respiratory rate to approximately 20 breaths per min	Monitoring and observation

BE (B) = base excess in blood, BP = blood pressure, HR = heart rate, Lac = lactate.

### 2.3. Interventions

1. Irrigation immediately stopped; operating table repositioned to 20° head-up.
2. Administered 100% oxygen via a non-rebreathing mask.
3. Mannitol 20% 200 mL IV over 15 minutes, furosemide 20 mg IV.
4. Dexmedetomidine 0.7 µg kg^−1^ h^−1^ infusion started; propofol 100 mg IV for seizure control.
5. Methylprednisolone 80 mg IV to attenuate cerebral edema.

### 2.4. Outcomes

Following recognition of irrigation-related intracranial hypertension, all interventions were started within 10 minutes. Generalized tonic-clonic seizures ceased 45 minutes after onset; the patient was fully orientated (Glasgow Coma Scale 15/15) 4 hours later. SBP returned to baseline 120 to 130 mm Hg without additional antihypertensives.

Arterial blood gas basically normalized within 3 hours (pH 7.21 → 7.53, lactate 10.8 → 4.4 mmol/L). No further sedative or antiepileptic doses were required.

Twenty-four-hour neurological examination: the National Institutes of Health Stroke Scale 0, the Mini-Mental State Examination 29/30, and negative meningeal signs. Mobilization: assistance on postoperative day (POD) 2, and independent ambulation on POD 5. Discharge: POD 7, modified Rankin Scale 0, no symptoms. Thirty-day telephone follow-up: modified Rankin Scale still 0, no headache, seizures, or cognitive change. Postoperative imaging was declined by the patient because of full clinical recovery.

## 3. Discussion

### 3.1. Pathophysiology of irrigation-related CSF hypertension

Continuous irrigation during PELD serves to clear debris and achieve hemostasis. When a dural tear occurs, pressurized fluid can enter the subarachnoid space, rapidly increasing intracranial volume. The Monro-Kellie doctrine dictates that compensatory mechanisms (CSF displacement, venous outflow) are exhausted within minutes when volume rises acutely.^[5]^ The Trendelenburg position further facilitates rostral fluid migration, exacerbating intracranial hypertension.

In our patient, the temporal relationship – refractory hypertension followed by immediate postoperative seizures – is consistent with this mechanism. The absence of preexisting intracranial pathology supports an acute iatrogenic etiology.

### 3.2. Differential diagnosis and misinterpretation pitfalls

Inadequate analgesia: hypertension is often attributed to surgical stimulation. However, escalating sevoflurane and remifentanil failed to attenuate SBP, suggesting an alternative cause.

VHM: VHM is characterized by chronic venous congestion and myelopathic signs.^[6]^ Our patient had no prior neurological deficits, and the acute presentation argued against VHM.

Postoperative delirium: Delirium typically manifests 24 to 72 hours postoperatively and is rarely associated with seizures.^[7]^

### 3.3. The uniqueness of this case

We compared and discussed 2 articles that meticulously documented severe complications occurring after PELD, and arrived at the following conclusions.

Our case aligns with earlier reports^[2,4]^ in which occult dural tears during PELD allowed pressurized irrigation to load the subarachnoid space, produce acute intracranial hypertension, and precipitate generalized tonic-clonic seizures. Like those patients, refractory hypertension was initially attributed to inadequate anesthesia, and rapid cessation of irrigation plus osmotic therapy secured full neurological recovery.

Novel contributions of the present report are the following:

1. Minute-by-minute arterial-line documentation that sustained hypertension preceded the seizure by 20 minutes, strengthening the cause–effect sequence.
2. First description of profound post-seizure lactic acidosis (pH 7.21, lactate 10.8 mmol/L), indicating either seizure-induced hypermetabolism or a systemic metabolic consequence of massive intraoperative irrigation.
3. Confirmation that refractory hypertension unresponsive to routine anesthetic adjustment is an early, actionable warning of impending seizure, a clinical cue underemphasized in previous literature.

### 3.4. Preventive strategies

#### 3.4.1. Surgical measures

1. Limit irrigation height ≤0.8 mH_2_O and flow ≤150 mL min^−1^.^[8]^
2. Warm saline to 37°C to reduce vasodilatory reflexes.
3. Meticulous surgical technique to minimize dural injury.

#### 3.4.2. Anesthetic measures

1. Continuous arterial pressure monitoring; consider intrathecal pressure transduction in high-risk cases.
2. Any intraoperative hypertensive surge >30% baseline unresponsive to anesthetic adjustment should prompt immediate cessation of irrigation and positional change.

### 3.5. Limitations

Direct measurement of intracranial pressure was not feasible, and postoperative imaging was not obtained due to patient agitation. As such, our interpretation is largely based on clinical observation and hemodynamic trends, which inevitably limit the objectivity of the findings. In addition, although the sequence of refractory hypertension and seizure followed irrigation closely, we cannot completely rule out other contributing factors, such as variations in anesthetic depth, individual drug response, or an underlying seizure tendency. Further cases, especially those with intraoperative neuromonitoring or postoperative imaging, would be valuable in clarifying the underlying mechanisms.

## 4. Conclusion

Irrigation-related CSF hypertension is a rare but reversible complication of endoscopic lumbar surgery. Vigilant hemodynamic monitoring and immediate cessation of irrigation when hypertension is refractory to anesthetic adjustment are paramount to preventing neurological injury.

## Acknowledgments

The authors thank the operating room of Qingdao Central Hospital for their meticulous documentation that made this report possible.

## Author contributions

**Conceptualization:** Yuxiang Wang, Rong Huang, Xiangjing Kong, Shoushi Wang.

**Data curation:** Yuxiang Wang.

**Formal analysis:** Yuxiang Wang, Yanyu Qi.

**Funding acquisition:** Yanyu Qi.

**Visualization:** Rong Huang.

**Supervision:** Xiangjing Kong.

**Writing – original draft:** Shoushi Wang.
